# Effects of K-115 (Ripasudil), a novel ROCK inhibitor, on trabecular meshwork and Schlemm’s canal endothelial cells

**DOI:** 10.1038/srep19640

**Published:** 2016-01-19

**Authors:** Yoshio Kaneko, Masayuki Ohta, Toshihiro Inoue, Ken Mizuno, Tomoyuki Isobe, Sohei Tanabe, Hidenobu Tanihara

**Affiliations:** 1 Tokyo New Drug Research Laboratories, Kowa Co., Ltd., Tokyo, 189-0022, Japan; 2 Department of Ophthalmology, Faculty of Life Sciences, Kumamoto University, Kumamoto, 860-8556, Japan

## Abstract

Ripasudil hydrochloride hydrate (K-115), a specific Rho-associated coiled-coil containing protein kinase (ROCK) inhibitor, was the first ophthalmic solution developed for the treatment of glaucoma and ocular hypertension in Japan. Topical administration of K-115 decreased intraocular pressure (IOP) and increased outflow facility in rabbits. This study evaluated the effect of K-115 on monkey trabecular meshwork (TM) cells and Schlemm’s canal endothelial (SCE) cells. K-115 induced retraction and rounding of cell bodies as well as disruption of actin bundles in TM cells. In SCE-cell monolayer permeability studies, K-115 significantly decreased transendothelial electrical resistance (TEER) and increased the transendothelial flux of FITC-dextran. Further, K-115 disrupted cellular localization of ZO-1 expression in SCE-cell monolayers. These results indicate that K-115 decreases IOP by increasing outflow facility in association with the modulation of TM cell behavior and SCE cell permeability in association with disruption of tight junction.

Rho-kinase (Rho-associated coiled-coil containing protein kinase; ROCK), a member of the serine-threonine protein kinases, is an effector protein of low molecular weight protein, Rho^1^. Rho kinase binds with Rho to form a Rho/Rho-kinase complex, and regulates various physiological functions, such as smooth muscle contraction, chemotaxis, neural growth, and gene expression^1^,^2^,^3^,^4^,^5^,^6^.

ROCK has two isoforms, ROCK-1 and ROCK-2, which are extensively distributed throughout in various tissues^7^. Both ROCK-1 and ROCK-2 are also widely-expressed in ocular tissues including the ciliary muscles, trabecular meshwork, iris, and retina, among others^8^. ROCK performs several physiological functions and aberrant regulation of ROCK levels has been shown to be involved in the pathogenesis of glaucoma, ocular hypertension, diabetic retinopathy, age-related macular edema, cataract, corneal dysfunction, and retinal disorders^9^,^10^,^11^,^12^,^13^,^14^,^15^,^16^,^17^,^18^. ROCK inhibitors have demonstrated efficacy in reducing intraocular pressure (IOP)^9^,^10^,^19^,^20^.

Glaucoma is primarily a disease affecting the optic nerve head that characteristically leads to visual field loss and ultimately blindness. Primary open-angle glaucoma (POAG), the commonest form of glaucoma, develops due to chronically elevated IOP as a result of pathologically increased resistance to the drainage of aqueous humor through outflow pathways^21^. IOP reduction is currently the only reliable, evidence-based management approach for the treatment of glaucoma^22^. Treatment strategies are decided according to glaucoma stage, type, and condition, with pharmacological agents considered the first-line therapy in most types of glaucoma^23^. Therapeutic agents for glaucoma include prostaglandin (PG) analogs, β-adrenergic receptor blockers, αβ-adrenergic receptor blockers, α1-adrenergic receptor blockers, α2-adrenergic receptor agonist, and carbonic anhydrase inhibitors. However, the reduction of IOP below target levels is often challenging with monotherapy^24^. PG analogs are often used as first-line therapy; however, there are concerns regarding adverse reactions, including eyelash changes and pigmentation changes affecting the iris and eyelid due to increased melanin production^25^. There is concern regarding the use of β-blockers due to potential effects on the cardiovascular (bradycardia) and respiratory (airway obstruction) systems^26^. Carbonic anhydrase inhibitors, often combined with first-line therapies, have been reported to cause ocular discomfort including irritation and blurred vision^27^. Consequently, there is a great clinical need for novel agents with potent IOP-lowering effects, without causing the adverse reactions described above. Furthermore, there is a need for pharmacological agents that can be used in combination with existing therapies.

K-115 (Ripasudil hydrochloride hydrate, 4-Fluoro-5-{[(2*S*)-2-methyl-1,4-diazepan-1-yl] sulfonyl} isoquinoline monohydrochloride dihydrate) is the first Rho-kinase inhibitor ophthalmic solution developed for the treatment of glaucoma and ocular hypertension in Japan^28^,^29^,^30^,^31^,^32^. In the previous study, the IOP-lowering effect of K-115 was induced by potentiation of the outflow facility from the conventional outflow route^29^. In this study, we examined the effects of K-115 on trabecular meshwork and Schlemm’s canal endothelial cell morphology *in vitro*.

## Results

### IOP-Lowering Effect of K-115 in Rabbits and Monkeys

**Figure 1:**
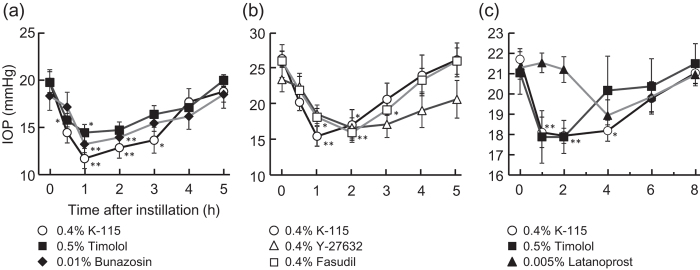
IOP-lowering effects of K-115. (**a**) Male albino rabbits were administered 50 μL of 0.4% K-115, 0.5% timolol, or 0.01% bunazosin into one eye (n = 9). (**b**) rabbits were administered 50 μL of K-115, Y-27632, or fasudil at concentrations of 0.4% into one eye (n = 6). The contralateral eye was not treated. IOP were measured using pneumotonometers prior to experiments and at 0.5, 1, 2, 3, 4, and 5 h after instillation. (**c**) Male cynomolgus monkeys were administered 20 μL of 0.4% K-115, 0.5% timolol, or 0.005% latanoprost into one eye (n = 5–6). The contralateral eye was not treated. IOP were measured using pneumotonometers prior to experiments and at 1, 2, 4, 6, and 8 h after instillation. All data are presented as mean ± SE. *P < 0.05, **P < 0.01, compared with preinstillation values (Dunnett’s multiple comparison test).

In rabbits, 0.4% K-115 ophthalmic solution demonstrated a significant IOP-lowering effect. Similar effects were observed by other glaucoma therapeutic agents (Fig. 1a). Furthermore, Y-27632 and fasudil showed similar IOP-lowering effect with K-115.(Fig. 1b). In monkeys, 0.4% K-115 ophthalmic solution showed a significant IOP-lowering effect. Additionally, an IOP-lowering effect of 0.4% K-115 was similar to existing agents (Fig. 1c).

### Effects of K-115, Y-27632, and Fasudil on TM Cell Morphology

**Figure 2:**
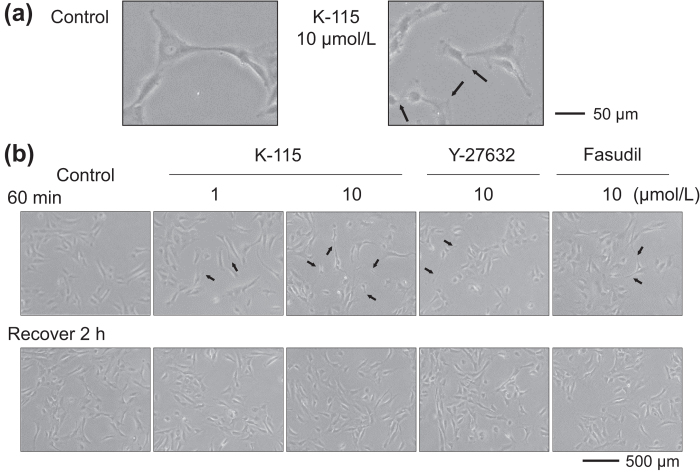
Effects of K-115 on cultured trabecular meshwork cell morphology. Phase-contrast microscopy of semiconfluent cultured TM cells. (**a**) Enlarged representative image of control (PBS) and 10 μmol/L of K-115 at 60 min after treatment. Scale bar: 50 μm. (**b**) Cells were incubated with 1 or10 μmol/L of K-115, 10 μmol/L of Y-27632, 10 μmol/L of fasudil, or PBS for 60 min. The addition of K-115, Y-27632, and fasudil was found to result in retraction and rounding of cells (black arrows). Drugs were removed afterward and replaced with culture medium. Recovery of normal morphology was observed 2 h after removal of each treatment agent. Scale bar: 500 μm.

**Figure 3:**
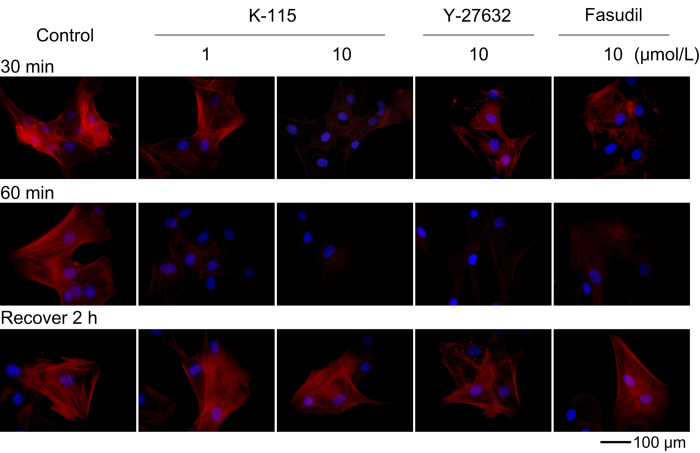
Changes in F-actin distribution with K-115 treatment of cultured trabecular meshwork cells. Distribution of F-actin (red) and DAPI (blue) in TM cells. Cells were incubated with 1 or 10 μmol/L of K-115 or 10 μmol/L of Y-27632, fasudil, or PBS (control) for 30 and 60 min. The addition of K-115, Y-27632, or fasudil resulted in the majority of cells losing the presence of actin bundles. Recovery of actin bundles was observed 2 h after the removal of each treatment agent. Scale bar: 100 μm.

The morphology of TM cells was examined. Treatment with 1 and 10 μmol/L of K-115 for 60 min was found to induce retraction and rounding of TM cells by light microscopy (Fig. 2). Similar changes were induced by 10 μmol/L of Y-27632 or fasudil. In TM cells, treatment with 1 and 10 μmol/L of K-115, 10 μmol/L of Y-27632, or 10 μmol/L of fasudil for 30 and 60 min reduced actin bundles (Fig. 3). Induced cytoskeletal changes, including retraction and cell rounding and reduced actin bundles, recovered 2 h after the removal of ROCK inhibitors. These data suggest that ROCK inhibition may initiate cytoskeletal rearrangement in TM cells.

### Effects of K-115, Y-27632, and Fasudil on SCE Cell Monolayer Barrier Function

**Figure 4:**
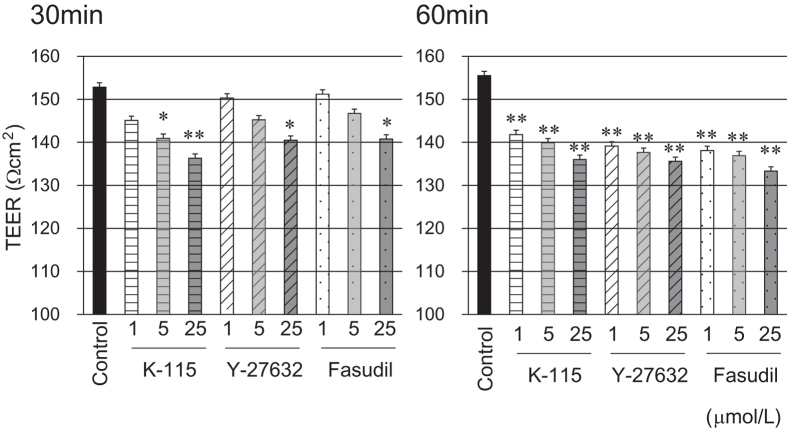
Effects of K-115, Y-27632, and fasudil on TEER in SCE cell monolayer. SCE cells were treated with K-115, Y-27632, or fasudil at concentrations of 1, 5, or 25 μmol/L for 30 and 60 min. All data are presented as mean ± SE (n = 6). *P < 0.05, **P < 0.01, compared to the control (Dunnett’s multiple comparison test).

**Figure 5:**
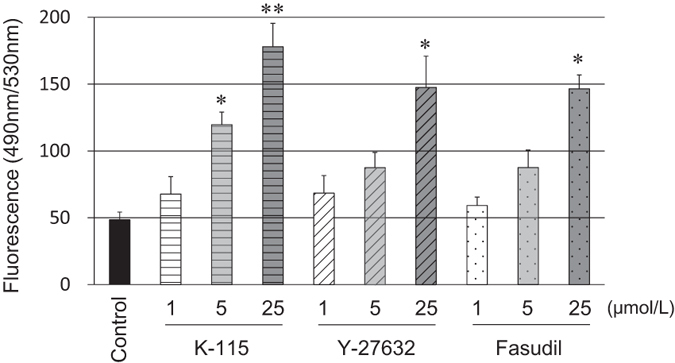
Effects of K-115, Y-27632, and fasudil on FITC-dextran permeability in SCE cell monolayers. SCE cells were treated with K-115, Y-27632, or fasudil at concentrations of 1, 5, or 25 μmol/L for 60 min. Data in each column are presented as mean ± SE (n = 6). *P < 0.05, **P < 0.01, compared to the control (Dunnett’s multiple comparison test).

To evaluate SCE cell monolayer barrier function, TEER (Fig. 4) and FITC-dextran permeability (Fig. 5) were measured. Treatment of SCE cell monolayers with 5 μmol/L of K-115, 25 μmol/L of Y-27632, or 25 μmol/L of fasudil resulted in significant reduction in TEER as 30 min. At 60 min, K-115, Y-27632, and fasudil were all found to significantly decrease TEER at concentration of 1 μmol/L. FITC-dextran permeability was significantly increased by treatment with 5 μmol/L of K-115 for 60 min. Similar effects were observed with 25 μmol/L of Y-27632 and fasudil. These results indicate that ROCK inhibitors reduce TEER and increase FITC-dextran permeability in a dose-dependent manner.

### Effect of K-115, Y-27632, and Fasudil on Molecules Associated with Cell–Cell Contact in SCE cells

**Figure 6:**
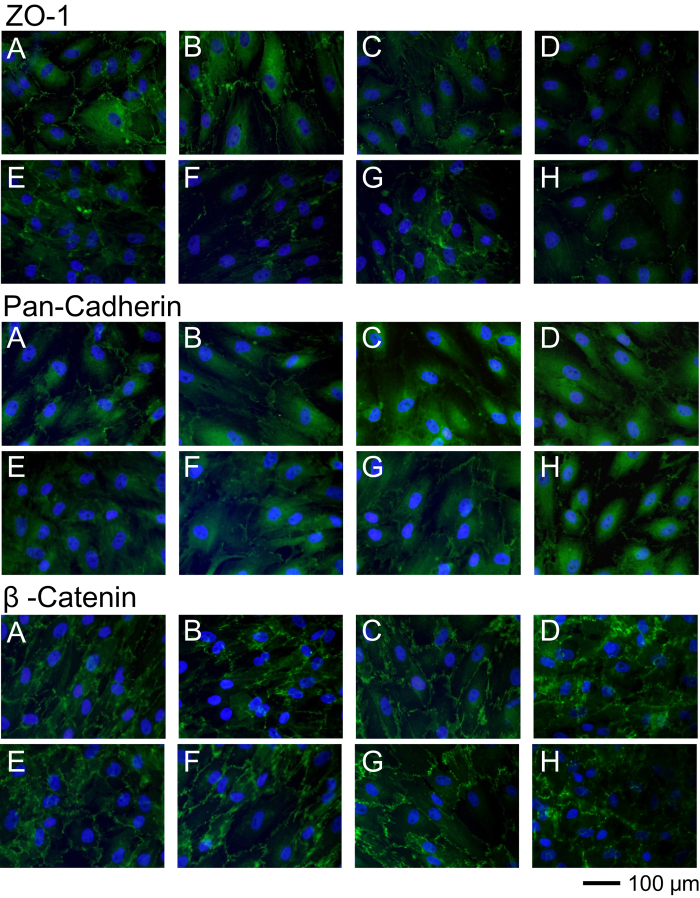
Effects of K-115 on cell–cell contact in SCE cells. Cultured SCE cells were treated with PBS (**A**), 1 (**B**), 5 (**C**), 25 μmol/L (**D**) of K-115, 5 (**E**), 25 μmol/L (**F**) of Y-27632, or 5 (**G**), 25 μmol/L (**H**) of fasudil for 60 min before immunostaining for cell–cell contact markers: ZO-1, β-catenin, and pan-cadherin (green). Cell nuclei were counterstained with DAPI (blue). Scale bar: 100 μm.

To examine cell junctions in SCE cells, a number of junctional complex proteins, including ZO-1, pan-cadherin, and β-catenin (Fig. 6), were evaluated by immunohistochemical analysis. Following treatment with 5, 25 μmol/L of K-115, 25 μmol/L of Y-27632, or 25 μmol/L of fasudil, areas stained for ZO-1 were decreased in SCE cells. However, any ROCK inhibitors did not change the localization of β-catenin and pan-cadherin. The changes in adherence junctions in response to ROCK inhibitors were less prominent, if present at all, compared to changes in tight junctions.

## Discussion

The effects of K-115 have previously been investigated in non-clinical studies. K-115 inhibit both human ROCK-1 (IC_50_ 0.051 μmol/L) and ROCK-2 (IC_50_ 0.019 μmol/L) more potently than the other ROCK inhibitors, Y-27632 and fasudil^29^. In the study of rabbits and monkeys with normal IOP, K-115 at a concentration of 0.4% that was a dose used in clinical showed IOP-lowering effect same as existing glaucoma therapeutic agents. In the previous study, a significant and dose-dependent IOP-lowering effect was observed with topical, unilateral, and single instillation of K-115 in rabbits and monkeys^29^. Furthermore, the ocular hypotensive effect of K-115 was immediate with the maximum effect (lowest IOP) close to the episcleral venous pressure^33^. These results indicate that K-115 is a highly potent and selective ocular hypotensive agent. In this study, same concentration of K-115, Y-27632 and fasudil showed significant IOP-lowering effect in rabbits. Similar results in rabbits have been reported with the single instillation of Y-27632, fasudil, and other ROCK inhibitors^19^,^20^,^34^,^35^. Therefore, the IOP-lowering effect of K-115 appears to be due to ROCK inhibition.

We previously investigated the effect of K-115 on the aqueous humor dynamics in rabbits (summarized in Table 1)^29^. Significant increases in outflow facility were observed after the instillation of K-115 at a concentration of 0.4%; however, we did not detect a significant effect of K-115 instillation on uveoscleral outflow or aqueous flow rate. These findings corroborate previous studies of other ROCK inhibitors, including Y-27632 and fasudil, in rabbits or monkeys^19^,^20^,^36^. These results strongly indicated that the ocular hypotensive effect of K-115 is potentiation of the outflow facility from the conventional outflow route.

The conventional pathway through the TM and Schlemm’s canal is the major route of aqueous humor outflow in primates^37^,^38^,^39^. The aqueous humor outflow resistance is generated in the inner wall of Schlemm’s canal and the juxtacanalicular region of TM in both normal and glaucomatous eyes^21^,^40^,^41^,^42^,^43^. Cytoskeletal structure properties, adhesive interactions, SCE cell permeability, and TM cell secretion are all proposed to play important roles in the regulation of aqueous humor outflow^37^,^44^,^45^. In the present study, we demonstrated the IOP-lowering mechanisms of K-115 by promoting aqueous outflow through the TM to Schlemm’s canal. In monkey TM cells, K-115 induced retraction and rounding, and reduced actin bundles. Additionally, these effects were reversible. Similar effects have been reported for Y-27632 and fasudil^19^,^20^,^46^. Cytoskeletal changes are thought to induce retraction and rounding of cells and reduce actin bundles in response to ROCK inhibition by K-115 which may lead to reduced compaction of TM tissue and increased aqueous outflow^46^. These changes may reduce resistance to fluid flow. The PKC inhibitor, GF109203X, has been reported to induce TM cell retraction and increase outflow facility in porcine eyes^47^. However, 1 μmol/L of K-115, enough to induce TM cell retraction, was found to have minimal inhibitory effect on PKC^29^. In addition, PKC inhibition by GF109203X only had an effect on outflow facility when infused into the anterior chambers, at much higher concentrations than found with topical administration of K-115. Furthermore, K-115 had no effects to other receptors and enzymes (data not shown) including serine-threonine protein kinases. These findings demonstrate K-115 induces cytoskeletal changes dependent on ROCK inhibition.

Schlemm’s canal is an important ocular component for outflow resistance against aqueous humor in the conventional outflow route with junctional protein complexes in SCE cells creating a barrier against aqueous humor outflow^48^,^49^. In the studies of the barrier function of monkey SCE cell monolayers, K-115 reduced TEER and increased FITC-dextran permeability. Furthermore, K-115 was decreased ZO-1 immunostaining areas in the SCE cells. However, K-115 did not change the localization of β-catenin and pan-cadherin. These results suggest that K-115 reduces outflow resistance and increases SCE cell permeability in association with tight junction disruption. Similar results were observed with the use of other ROCK inhibitors, Y-27632^48^. In addition, low concentrations of K-115 induced similar potential for monolayer cell permeability compared with higher concentrations of Y-27632 and fasudil. Therefore, promotion of aqueous outflow by K-115 is likely due to TM cytoskeletal changes, reduced outflow resistance, and increased SCE permeability as a result of ROCK inhibitory activity. Furthermore, Fujimoto *et al*. reported that the ROCK inhibitor, Y-27632, improved dexamethasone-induced reduction of the outflow facility in porcine eyes and increased TEER in SCE cells^50^. These results suggest that ROCK was concerned strongly to hypertension induced by dexamethasone. Therefore, the ROCK inhibition by K-115 may be efficient in reducing IOP in glucocorticoid-induced ocular hypertension.

In this study, we demonstrated that topical instillation of the novel and selective ROCK inhibitor, K-115, decreased IOP and increased conventional aqueous outflow by altering TM cell morphology and the permeability of SCE cells. An ophthalmic solution of K-115 is the first Rho-kinase inhibitor developed for the treatment of glaucoma in Japan. It exhibits an IOP-lowering effect through the promotion of aqueous outflow through the TM to Schlemm’s canal. As K-115 has a different mechanism of action from the agents that suppress aqueous humor production, including β-adrenergic receptor blockers, carbonic anhydrase inhibitors, and α2-adrenergic receptor agonists, and agents that promote uveoscleral outflow, including α1-adrenergic receptor blockers, α2-adrenergic receptor agonists, and prostaglandin analogues. K-115 is expected to have substantial utility in providing a concomitant effect with existing drugs or providing a greater variety of pharmacological treatment options for glaucoma.

## Methods

### Animals

Male Japanese white rabbits weighing 2.0–3.0 kg and male cynomolgus monkeys weighing 2.5–5.0 kg (3 years or older) were used in this study. All studies were conducted in accordance with the ARVO Statement for the Use of Animals in Ophthalmic and Vision Research and were approved by the Animal Ethics Committee of Kowa Tokyo New Drug Research Laboratories. The animals were housed in an air-conditiond room (23 ± 2 °C, 50 ± 10% humidity) lit from 7:00-19:00, and were allowed food and water *ad libitum* throughout the experiments.

### Chemicals and Drug Preparation

K-115 was synthesized at Tokyo New Drug Research Laboratories, Kowa Co. Ltd. (Tokyo, Japan). Y-27632 and fasudil were purchased from Calbiochem (San Diego, CA) and Enzo Life Sciences (Farmingdale, NY), respectively. For topical instillation studies, K-115, Y-27632 and fasudil were dissolved in a vehicle containing preservative, for clinical use as an ophthalmic solution. 0.5% timolol (Timoptol^®^ Ophthalmic Solution 0.25%; Santen Pharmaceutical Co. Ltd.), 0.01% bunazosin (Detantol^®^ 0.01% ophthalmic solution; Santen Pharmaceutical Co. Ltd.) and 0.005% latanoprost (Xalatan^®^ Eye Drops 0.005%; Pfizer Inc, Tokyo, Japan) were used commercial formulation.

### Effect of Topical K-115 Administration on Intraocular Pressure

Pneumotonometers (Model 30 Classic Pneumotonometer; Medtronic Solan Ophthalmic Products Inc., Jacksonville, FL) were used to monitor IOP. For IOP measurements, eyes were anesthetized by topical instillation of 0.4% oxybuprocaine (0.4% Benoxil^®^ ophthalmic solution; Santen Pharmaceutical Co. Ltd., Osaka, Japan). In albino rabbit experiments, 50 μL of 0.4% K-115, 0.5% timolol, 0.01% bunazosin, 0.4% Y-27632 or 0.4% fasudil ophthalmic solution were instilled into one eye. The contralateral eye was not treated. IOP was measured in both eyes prior to the experiment and at 0.5, 1, 2, 3, 4, and 5 h after instillation.

In the monkey experiments, 20 μL of 0.4% K-115, 0.5% timolol or 0.005% latanoprost ophthalmic solution were instilled into one eye. The contralateral eye was not treated. IOP was measured in both eyes prior to the experiment and at 1, 2, 4, 6, and 8 h after instillation. IOP were compared between pre-instillation and each measured time point after drug instillation.

### Monkey TM and SCE Cell Culture

Monkey TM and SCE cells were isolated from eyes of cynomolgus monkeys (6 to 12 months old) obtained from Shin Nippon Biomedical Laboratories (SNBL; Kagoshima, Japan) according to a previously described method^48^,^50^,^51^,^52^. Schlemm’s canal was identified by luminal cannulation with a 6–0 nylon suture under microscopic observation. TM tissues were manipulated with fine forceps and placed on collagen gel-coated plates. After complete removal of TM tissues, explants of the inner wall of Schlemm’s canal were placed on collagen gel-coated plates. Primary TM and SCE cells were expanded in Dulbecco’s modified Eagle medium (DMEM; WAKO Pure Chemical Industries, Osaka, Japan) supplemented with 10% fetal bovine serum (FBS; Hyclone Laboratories, Logan, UT), 2 mmol/L glutamine, 100 U/mL penicillin, 100 μg/mL streptomycin, and 0.5 μg/mL amphotericin B at 37 °C in 5% CO_2_. TM cells between passages 4 and 5, and SCE cells between passages 4 and 8, were used in this study.

### Effects of K-115, Y-27632, and Fasudil on TM Cell Morphology

The effect of the K-115 on cell morphology was investigated according to previously described methods^19^,^20^. TM cells were plated on 6 well plates at a density of 1 × 10^4^ cells per well in DMEM containing 10% FBS. Following overnight culture, when cells had reached semiconfluence, 1 or 10 μmol/L of K-115, 10 μmol/L of Y-27632, or 10 μmol/L of fasudil were added to culture wells. PBS was used as a control vehicle. After 60 min, drug solutions were removed and replaced with DMEM containing 10% FBS. Cells were observed by phase-contrast microscopy and photographed 60 min after drug application and 2 h after drug removal. For immunohistochemistry, TM cells were plated on gelatin-coated 8 well chamber slides at a density of 1 × 10^4^ cells per well in DMEM containing 10% FBS. After overnight culture, when cells reached semiconfluence, cell were incubated in K-115 at 1 or 10 μmol/L, Y-27632 at 10 μmol/L, or fasudil at 10 μmol/L for 60 min. PBS was used as a control vehicle. Drug solutions were removed and replaced with DMEM containing 10% FBS after 2 h. Cells were fixed with 4% paraformaldehyde in PBS for 15 min then washed with cytoskeletal buffer (10 mmol/L MES [2-morpholinoethansulfonic acid potassium salt], 150 mmol/L NaCl, 5 mmol/L EGTA, 5 mmol/L MgCl_2_, 5 mmol/L glucose, pH 6.1) and serum buffer (10% FBS in PBS). Cells were permeabilized with 0.5% Triton X-100 in PBS for 12 min at room temperature and blocked with serum buffer for at least 2 h at 4 °C. Filamentous actin (F-actin) was labeled with 0.05 mg/mL Phalloidin-TRITC (Sigma-Aldrich) for 1 h at room temperature. After washing with PBS, cells were mounted with commercial mounting medium (VECTASHIELD; Vector Laboratories, Burlingame, CA) containing 4’, 6-diamidino-2-phenylindole (DAPI) and observed using a fluorescence microscope (BX51; Olympus, Tokyo, Japan). The exposure to take images for F-actin and DAPI were 0.1 and 0.05 sec, respectively.

### Measurement of SCE Cell Monolayer Transendothelial Electron Resistance

SCE cell monolayer TEER was determined according to previously described methods^48^,^50^. SCE cells were seeded at 5 × 10^4^ cells/well and grown to confluence on polyester membrane inserts on 12-well culture plates (0.4 μm pore size and 12 mm diameter; Corning Transwell, Sigma-Aldrich) in DMEM supplemented with 10% FBS at 37 °C in 5% CO_2_. Apical side (inside of the membrane inserts) volumes were 0.5 mL and basal side (outside of the membrane inserts) volumes were 1.5 mL. Two weeks after seeding, TEER was measured. Culture medium was changed to DMEM supplemented with 1, 5, or 25 μmol/L of K-115, Y-27632, or fasudil. PBS was used as a control vehicle. TEER was measured using an electrical resistance system (Millicell ERS; Millipore, Billerica, MA) according to the manufacturer’s instructions at 30 and 60 min after drug treatment and recorded as net values (Ωcm^2^).

### Measurement of SCE Cell Monolayer Permeability

SCE cells were prepared by the same method used for TEER measurements as described above. SCE cell monolayers were then stimulated with 1, 5, or 25 μmol/L of K-115, Y-27632, or fasudil. A tracer, fluorescein isothiocyanate (FITC)-dextran (average molecular weight, 4000; Sigma-Aldrich), was simultaneously applied at 50 μmol/L to culture well basal compartments. Culture medium was collected from the apical side for fluorescence measurements at 60 min after the addition of tracer with an equal volume of the culturing medium added to replace the removed medium. FITC-dextran concentrations in collected medium were measured using a multimode plate reader (Gemini XPS; Molecular Devices, LLC, Sunnyvale, CA) with an excitation wavelength of 490 nm and an emission wavelength of 530 nm. The fluorescence intensity of normal medium was measured and used as the background concentration in each experiment.

### SCE Cell Immunofluorescence Microscopy

SCE cells were cultured on collagen-coated 8 well chamber slides at a density of 1 × 10^4^ cells per well in DMEM containing 10% FBS. Following overnight culture, when cells had reached confluence, K-115 at 1, 5, or 25 μmol/L, Y-27632 at 5 or 25 μmol/L, or fasudil at 5 or 25 μmol/L were added to culture media and cells were incubated for 30 min. PBS was used as a control vehicle. Cells were fixed with 4% paraformaldehyde in PBS for 15 min then washed with cytoskeletal buffer and serum buffer. Cells were permeabilized with 0.5% Triton X-100 in PBS for 12 min at room temperature. After washing in serum buffer, cells were blocked with serum buffer for at least 2 h at 4 °C. Cells were incubated overnight at 4 °C with the following primary antibodies: rabbit anti-ZO-1 (1.25 μg/mL; Invitrogen, Carlsbad, CA), anti-β-catenin (1:1000 dilution; Sigma-Aldrich), and anti-pan-cadherin (1:100 dilution; Sigma-Aldrich). Cells were washed in serum buffer and then incubated with secondary antibody (Alexa Fluoro 488; Invitrogen) for 30 min at room temperature. After washing in PBS, cells were mounted with mounting medium contained DAPI and observed using a fluorescence microscope. The exposure to take images for ZO-1, β-catenin, pan-cadherin and DAPI were 0.5, 0.5, 0.8 and 0.05 sec, respectively.

### Statistical Analyses

All data are expressed as mean ± SEM unless indicated otherwise. For IOP measurements, SCE cell monolayer TEER, and cell permeability studies, statistical analyses were conducted using either one-way analysis of variance (ANOVA) with repeated measures followed by the Dunnett’s test (two-tailed). *P* < 0.05 was predetermined as the criterion of statistical significance.

## Additional Information

**How to cite this article**: Kaneko, Y. *et al*. Effects of K-115 (Ripasudil), a novel ROCK inhibitor, on trabecular meshwork and Schlemm's canal endothelial cells. *Sci. Rep*. **5**, 19640; doi: 10.1038/srep19640 (2015).

## Figures and Tables

**Table 1:** Effects of K-115 on aqueous humor dynamics (summary of previous studies^29^).

	Outflow facility (C: μL/min/mmHg)	Uveoscleral Outflow (Fu: μL/min)	Aqueous Flow Rate (f(t): μL/min)
Vehicle	0.086 ± 0.021	0.134 ± 0.026	1.54 ± 0.23
K-115	0.193 ± 0.038	0.155 ± 0.023	1.88 ± 0.35
Student’s t-test	P = 0.032	NS	NS
Fold change	2.24	1.16	1.22

All data are presented as mean ± SE (n = 6–8) NS: not significant.
